# Improving the Error of Time Differences of Arrival on Partial Discharges Measurement in Gas-Insulated Switchgear

**DOI:** 10.3390/s18114078

**Published:** 2018-11-21

**Authors:** Jun Jiang, Kai Wang, Chaohai Zhang, Min Chen, Hong Zheng, Ricardo Albarracín

**Affiliations:** 1 Jiangsu Key Laboratory of New Energy Generation and Power Conversion, Nanjing University of Aeronautics and Astronautics, Nanjing 211106, China; wangkai7096@163.com (K.W.); zhangchaohai@nuaa.edu.cn (C.Z.); 2 State Grid Zhejiang Electric Power Co. Ltd. Research Institute, Hangzhou 310014, China; ncepucm@163.com; 3 Hangzhou Kelin Electric Power Equipment Co., Ltd., Hangzhou 310014, China; zhenghong@klec.com.cn; 4 Departamento de Ingeniería Eléctrica, Electrónica, Automática y Física Aplicada, Escuela Técnica Superior de Ingeniería y Diseño Industrial, Universidad Politécnica de Madrid, Ronda de Valencia 3, 28012 Madrid, Spain; ricardo.albarracin@upm.es

**Keywords:** partial discharge, Ultra-High-Frequency, Gas-Insulated Substations Fault location, Time difference of Arrival (TDoA)

## Abstract

Partial Discharge (PD) detection based on Ultra-High-Frequency (UHF) measurements in Gas-Insulated Switchgear (GIS) is often used for fault location based on extraction of Time Differences of Arrival (TDoA), and the core technique is to obtain the precise time difference of each UHF signal. Usually, TDoA extraction algorithms can be categorized as cross-correlation function method (CCF), minimum energy method (ME), and threshold value method (TV) are not qualified to analyze the time difference with high accuracy and efficiency, especially the complicated UHF PD signals in the field. In this paper, multiple tests were carried out based on the real GIS UHF signals. Three typical algorithms (CCF, ME, and TV) were used to extract and calculate the TDoA of UHF signals. Afterwards, depending on the disassembly of equipment, the accuracy and effective range of the algorithms are analyzed by means of error and variance. To minimize the error and the variance, an average method with the combination (CA) and portfolio of traditional algorithms is proposed and verified in different situations. The results demonstrate that the improved algorithm could increase the accuracy of time difference extraction, less than 4.0%.

## 1. Introduction

Gas-Insulated Switchgear (GIS) substations are widely used in power system due to the advantages of compact structure and free from external electromagnetic (EM) interference compare with Air-Insulated Substation (AIS) [1,2]. However, disassembling the devices to detect the location of PD costs a lot of time and efforts [3,4,5,6]. PD detection is usually taken as an important reference index in the insulation detection of GIS due to it being a good way to reflect location information of the GIS insulation fault [7,8,9,10]. The detection of PD can be carried out by Ultra-High-Frequency (UHF) method. It analyzing the UHF signals of two or more channels, the Time Differences of Arrival (TDoA) can be extracted and the fault point position can be calculated [11,12,13]. Among them, how to obtain TDoA accurately is the core of UHF method.

The classic TDoA extraction algorithms include cross-correlation function method (CCF), minimum energy method (ME) and threshold value method (TV). Nowadays, many emerging algorithms derive from these. Hou H. proposed four-order cumulant and bispectrum algorithms based on minimum energy method. It overcame the effect of Gaussian noises with unknown correlation [14]. Yang J. A. has improved the cross-correlation function algorithm in his thesis based on the wavelet transform and cubic splines interpolation which greatly eliminated the effects of background noise [15]. Li X. applied cubic spline interpolation to increase points between two real sample points and optimized cross-correlation function method. It makes initial peak wave easily distinguished [16]. In [14,15,16], it mainly improves the typical algorithm, but they were not verified and demonstrated through the measurement in field application. Threshold value method is adopted in Reference [17]; it was adopted in actual situation without considering how to improve the accuracy by perfecting the algorithm.

To promote the accuracy of PD location, an improved TDoA extraction algorithm is proposed. To detect PD fault, sensor arrangement was carried out based on real GIS in this manuscript. After getting UHF signals and using wavelet transform to remove environmental noise in UHF, three typical algorithms are used to extract TDoA [18]. By disintegrating GIS, the advantages, disadvantages and application scope of ME, CCF, and TV had been analyzed and contrasted. Furthermore, based on three typical algorithms were compensated and combined to minimize the measurement error and variance. Finally, it had been verified that the improved algorithm (Combination-Average, CA) could meet the needs of the TDoA extraction in the field.

## 2. Experimental Setup

**Figure 1:**
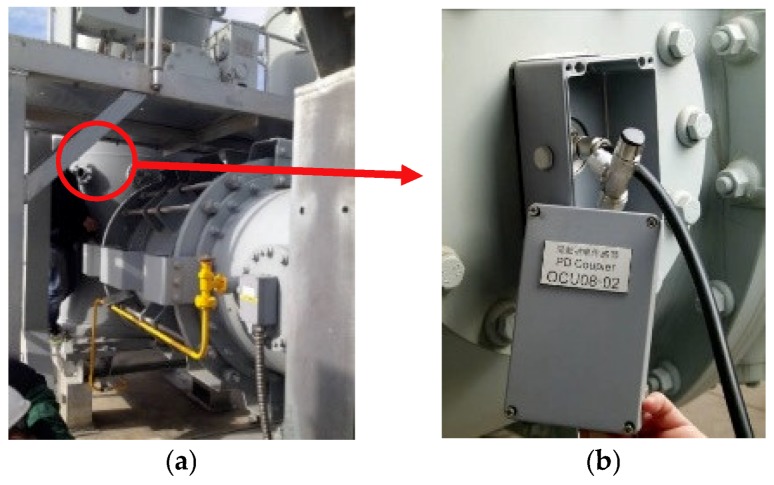
Installation of Ultra-High-Frequency (UHF) sensors on 1100 kV Gas-Insulated Switchgear (GIS) in the field. (**a**) Overall installation; (**b**) Partial details.

**Figure 2:**
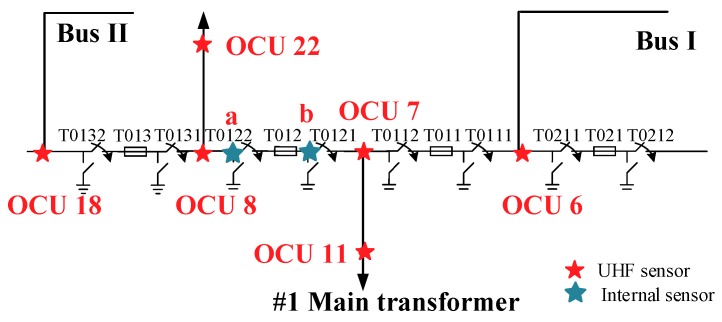
Layout of online monitoring system layout diagram.

Extraction TDoA from UHF signal is often used because of the fault location of GIS can be found quickly. Since the TDoA method is used to calculate the position of the PD, at least two UHF sensors must be measuring at the same time [19]. In order to meet the requirements, the measurements were carried out in a real installation, as shown in Figure 1. Operational Control Unit (OCU) was installed in the observation window of the disconnector and UHF sensors extract signals from OCU numbered 6, 7, 8, 11, 18, and 22, and a and b are sensors which extract signals from observation windows of T0121 and T0122. As shown in Figure 2, T0132, T013, T0131, T0122, T012, T0121, T0112, T011, T0111, T0211, T021, and T0212 are three-phase switching equipment, including circuit breakers, and the corresponding disconnectors and current transformers. OCU6, OCU7, OCU8, and OCU18 are separated by 60 m from each other, distance between a and b, OCU7 and OCU11, OCU8, and OCU22 is 30 m. The model of UHF sensors is a diagnostic monitoring system (DMS, AE01676) and the working frequency band of the UHF sensors locate in 300 MHz–2 GHz, and the experimental platform adopted LeCroy oscilloscope (U.S.), where sampling frequency is 10 G Sa/s. At the same time, to make the waveform smooth, the sampling frequency was increased 16 times by adding the sampling point between each two sampling points through interpolation calculation. Furthermore, the arrival time of the initial peak wave and the TDoA between two UHF signals are analyzed.

**Figure 3:**
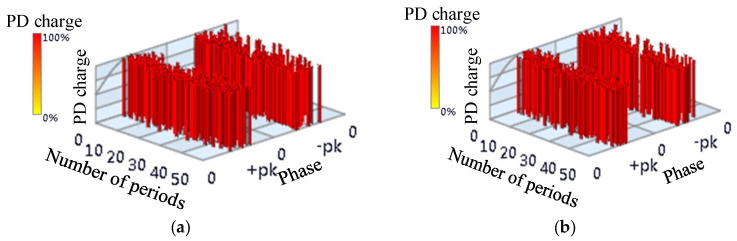
Phase-Resolved Pulse Sequence of the real Partial Discharge (PD) activity in Gas-Insulated Switchgear (GIS).

There is a real PD activity in GIS. Phase-Resolved Pulse Sequence (PRPS) of PD activity in GIS is shown as Figure 3, in which, X scale is phase, Y scale is number of periods, and Z scale shows the charge of the PD. For the sake of statistics, taking the maximum charge as a reference, the charge of partial discharge was normalized. It was expressed as percentage. UHF signals are extracted from OCU7 and OCU11. The phase of the signal has the characteristics of bipolarity and randomness, and the discharge signal had a relatively large amplitude [20,21,22], thus the type of discharge is floating discharge.

In order to facilitate the extraction of TDoA, identify the location of PD detection and consider noise, the TDoA of the initial peak wave was defined at first [23,24]. The absolute value of UHF signal amplitude is calculated to obtain a new discrete signal X(t), as Equation (1).

(1) X(t)=|U(t)|

**Figure 4:**
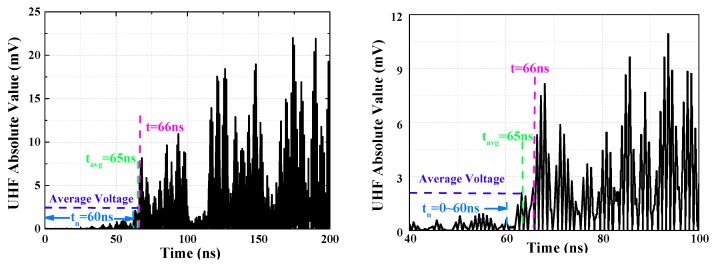
Schematic definition of the initial peak wave.

Average voltage can be acquired after averaging the discrete signals of the group. The time of absolute UHF signal firstly reaching the average voltage is defined as tavg. As UHF signal of PD reaches peak value rapidly, signal in which the time between *t* = 0 ns and (t=tn=tavg−5) ns is regarded as noise. Maximum amplitude of the noise after amplified 1.1 times is seen as the threshold value, the first crest that exceeds threshold value is defined as initial peak wave, and the time is arrival time of the initial peak wave. The definition of the initial peak wave is illustrated by taking the UHF signal collected by OCU7 as an example, as shown in Figure 4.

## 3. TDoA Extraction

Before the time difference is extracted, the waveform is firstly processed with the Matlab wavelet toolbox. Due to dB2 function is not easy to result in the boundary problem in the de-noising process and affect the accuracy of TDoA extraction, it is used as the wavelet de-noising function. The waveform after de-noising at different scales was compared, and the waveform with no distortion and maximum de-noising was selected as the waveform to extract TDoA. There are three typical methods of TDoA extraction.

### 3.1. Cross Correlation Function Method (CCF)

CCF can reflect the relationship between two random variables at different time. x(t), y(t) are samples obtained, respectively, by test, after the time of y(t) moving τ, the sample is defined as y(t+τ). The essence of TDoA estimation is to calculate the correlation of sequence x(t) and y(t). The CCF is defined as Equation (2):

(2) $$R_{xy}(\tau )=\underset{T\to \infty }{\lim}\frac{1}{2T}\int_{-T}^{T}x(t)y(t+\tau )dt$$

T is the total sampling time, and Rxy(τ) represents the cross-correlation extent. By simplification, the equation can be obtained:

(3) $$R_{xy}(m)=\underset{T\to \infty }{\lim}\frac{1}{2N+1}\sum_{k=-N}^{N}x(k)y(k+m)$$

N is number of sampling points. The time of CCF maximum peak represents the time difference between two samples. The time difference is:

(4) Δt=mTs

m is the number of sampling point where the maximum peak value of the cross-correlation function at, and Ts is the sampling time, unit is Sa/s.

**Figure 5:**
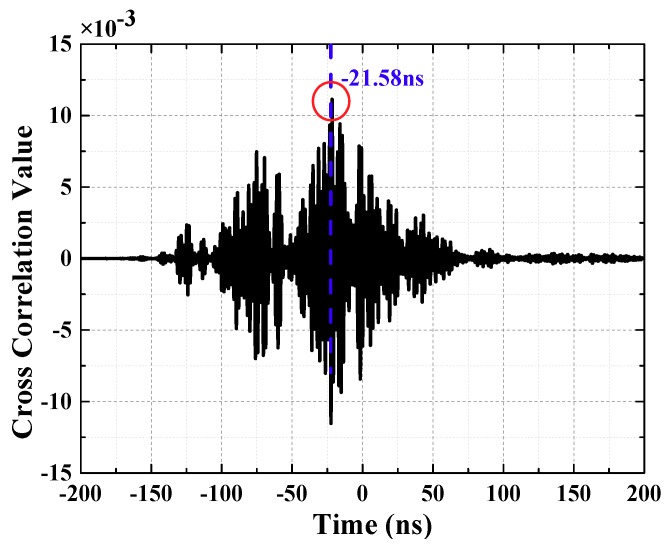
Waveform of cross-correlation function.

The calculation results are shown in Figure 5 (waveforms have been denoised by wavelet transform). Time difference obtained is −21.58 ns. Due to the essence of the Cross-correlation Function method is a set of signals was shifted in the time domain, it is obvious that the amount of signal translation is the TDoA between two UHF signals. If TDoA is positive, the moving UHF signal arrives ahead of another set of signals. On the contrary, it delays another UHF signal. Negative value indicated that UHF2 signal lags UHF1 signal. Thus, it is demonstrated that UHF2 lags UHF1 signal in Figure 5.

### 3.2. Minimal Energy Method (ME)

From the starting time of signal collection, the cumulative energy of UHF signals at each moment is calculated. The cumulative energy is:

(5) $$W(t)=\int_{t_{i}}^{t}\frac{u^{2}(t{}^{\prime })}{R}dt{}^{\prime }$$

$t_{i}$ (ns) is starting time of signal collection, R (Ω) is input impedance of collection system. $u(t{}^{\prime })$ is voltage of every sampling instant, the unit is V. The accumulated energy before the PD signal arrives is small and it rise slowly. The accumulated energy after the PD signal arrives is enormous and it rises rapidly. Therefore, there is an inflection point in the energy curve, which can reflect the arrival time of the initial peak wave. To facilitate the calculation, the mathematical derivation shows that the inflection point can be converted to the minimum point, this Equation is:

(6) $$W{}^{\prime }(t)=\int_{t_{i}}^{t}\frac{u^{2}(t{}^{\prime })}{R}dt{}^{\prime }-\frac{t-t_{i}}{T_{s}}\int_{t_{i}}^{t_{0}}\frac{u^{2}(t{}^{\prime })}{R}dt{}^{\prime }$$

$t_{0}$ is sampling end time. The energy curve obtained from the above equation is called the minimum energy curve. The minimum point of the curve corresponds to the moment of inflection point, which indicates the moment that the signal arrives.

The local maximum or minimum of a function as shown in Equation (7).

(7) $$\frac{dW{}^{\prime }(t)}{dt}=\frac{u^{2}(t)}{R}-\frac{1}{T_{s}}\int_{t_{i}}^{t_{0}}\frac{u^{2}(t{}^{\prime })}{R}dt{}^{\prime }=0$$

However, in practical application, most of the zeros obtained by calculating, always between two sampling points. Therefore, the method of acquiring extreme points is changed to the definition method. It means that the extreme point is between $\frac{dW{}^{\prime }(t)}{dt}<0$ and $\frac{dW{}^{\prime }(t)}{dt}>0$.

**Figure 6:**
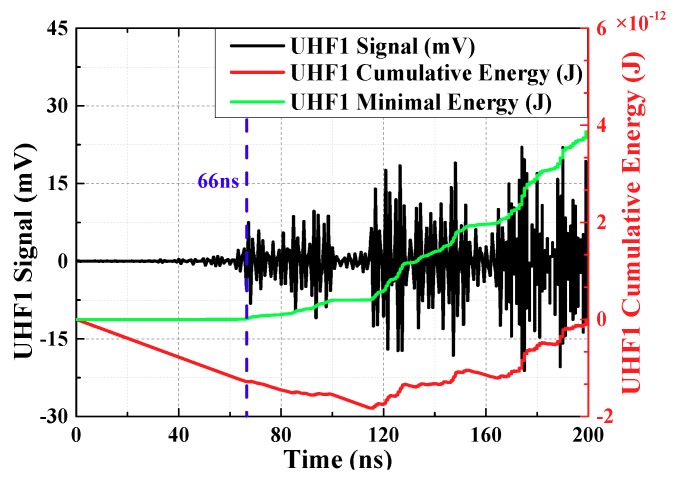
The curve about minimum energy method of waveform 1.

**Figure 7:**
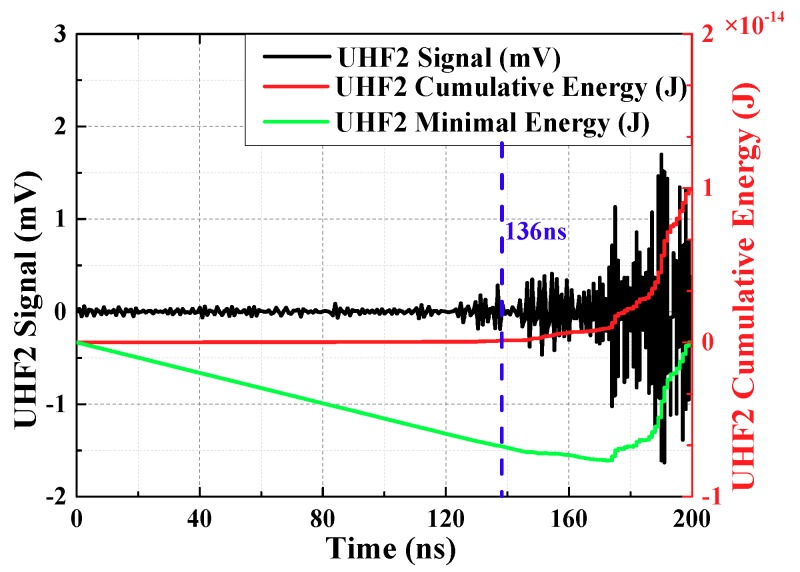
The curve about minimum energy method of waveform 2.

UHF1 signal had been extracted by OCU7, UHF2 signal had been extracted by OCU11. The calculation results are shown in Figure 6 and Figure 7 (waveforms have been denoised by wavelet transform). Time difference obtained is 70.59 ns.

### 3.3. Threshold Value Method (TV)

**Figure 8:**
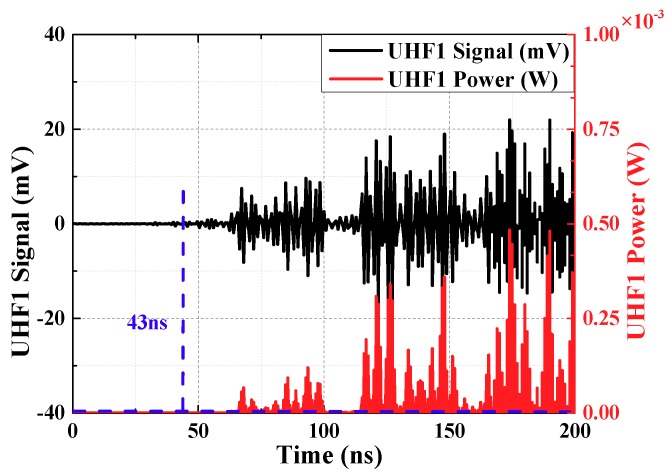
The curve about threshold value method of waveform 1.

**Figure 9:**
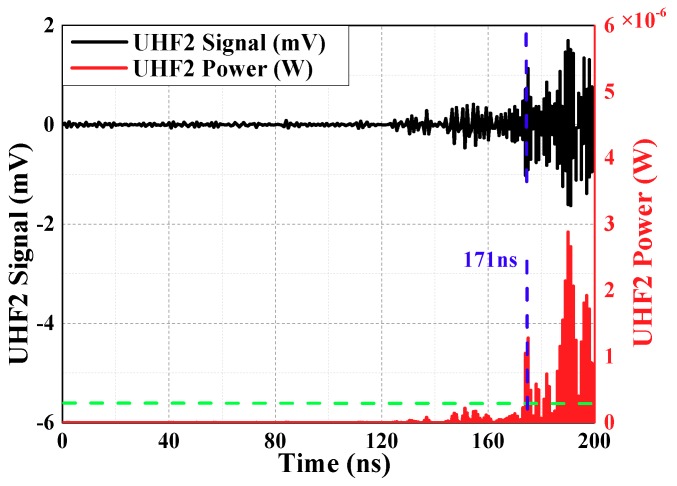
The curve about threshold value method of waveform 2.

The threshold value is set between the maximum noise value and the minimum PD signal value, and compares the absolute value of the UHF signal amplitude at each moment with threshold value, and the moment when the threshold value is exceeded first by the arrival time of the initial peak wave. To improve the signal to noise ratio (SNR), the signal voltage amplitude is converted into the power amplitude for calculation, as shown in Equation (8).

(8) $$P(t)=\frac{u^{2}(t)}{R}$$

u(t) is the same as above, is voltage of every sampling instant, the unit is V and it is calculated by choosing a suitable threshold value (it is twice maximum amplitude of the noise, maximum amplitude of the noise has been defined before). Calculation results are shown in Figure 8 and Figure 9 (waveforms have been denoised by wavelet transform). Time difference obtained is 128.03 ns.

## 4. Data Analysis

By selecting two different locations (randomly selected from the OCU) of UHF sensor signals, the results are shown in Table 1. The data reveals that it has extreme difference between TDoA are extracted by three typical algorithms.

**Figure 10:**
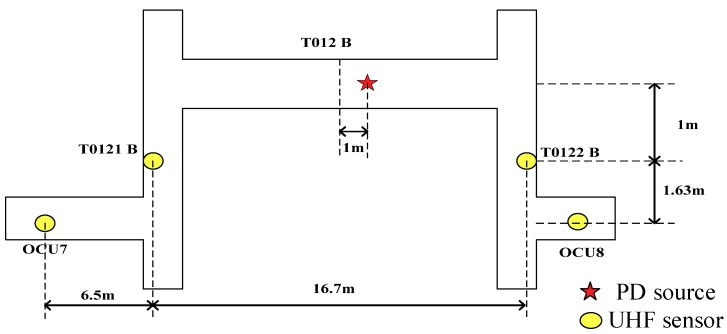
Fault location of PD activity in GIS.

When using the same set of UHF signals for TDoA extraction, TDoA extracted by different algorithms have great disparities, and TDoA were averaged to ensure that the error of one algorithm is too large to find the PD activity. Then, PD was located between a and b by UHF location, and thus, T012 had been disintegrated, as shown in Figure 10.

While the typical algorithm located the PD position, TDoA obtained by the three typical algorithms is different and the precision is relatively low, so distances from PD source to UHF sensors are converted into times, which were used as the reference to analyze the three typical algorithms. Data analysis results are shown in Table 2.

The cumulative energy inflection point is difficult to be misjudged. The ME method has the lowest average error, the smallest variance and the closest reference time difference when compared with TV and CCF. However, since the energy inflection point depends on when the energy exceeds the average energy, and the initial peak wave energy is less than the average energy, and the energy inflection point is not obvious, so TDoA is small. The voltage amplitude of the UHF signal obtained from the remote sensor is relatively small. After the wavelet de-noising, the average energy of previous initial peak wave arrived is extremely small, and therefore, the error of UHF signal, which remove the PD that is smaller than the error of UHF signal near the PD. Time difference extracted by the cumulative energy method is always less than the reference time difference in general, as shown in Table 2.

As the sample is a GIS, with a simple structure and UHF waveform, the impact of shell structure of high-voltage equipment is weak. The degree of electromagnetic distortion is very small, so the accuracy of threshold value method is relatively high and is closely related to the threshold selection. In this manuscript, the threshold value is two times greater than the noise signal of UHF. While the voltage had been converted to power, the SNR is still low. As a result, when TV extracts the TDoA, TV is easy to locate by mistaking the fluctuation signal, which reduces the detection accuracy, especially in the case of short-time difference (according to the experimental results, it is about 55 ns and below), the detection accuracy is low. Sensors that are near to the PD source are susceptible to fluctuating noise and therefore the initial peak wave time of extraction arrives too quickly, the UHF signals collected by sensors far away from the PD source only fluctuate violently when the PD is severe. Therefore, TV is more accurate to extract the arrival time of the initial peak wave in long TDoA.

The CCF is bound up with the shape of UHF waveform, and the error source is mainly from the incomplete waveform collected by the remote sensor. As shown in Table 2, the shorter TDoA is, the more similar waveform is. Due to this, the short TDoA will cause high accuracy in the CCF method.

According to the above analysis, although errors of three typical algorithms are different, but all of them are big. In order to improve the extraction accuracy of TDoA, the algorithm needs to be modified.

## 5. Improved Extraction Algorithm

**Figure 11:**
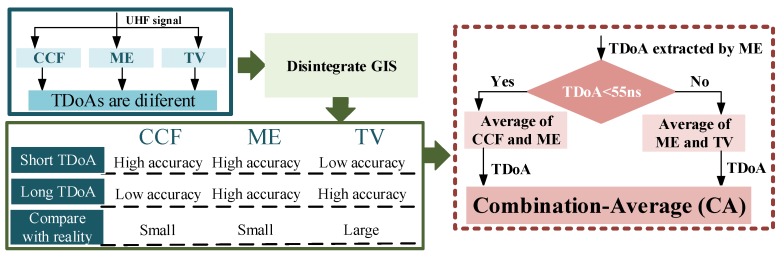
Algorithm interpretation of CA.

The disintegration of GIS demonstrated that CCF has high accuracy in the case of short TDoA. In this case, TV has low accuracy. The TDoA obtained by ME is relatively small when compared with the reference TDoA, and the TDoA of TV is larger. Therefore, the CA method can be used to improve the accuracy of TDoA extraction. Algorithm interpretation of CA is shown as Figure 11.

**Figure 12:**
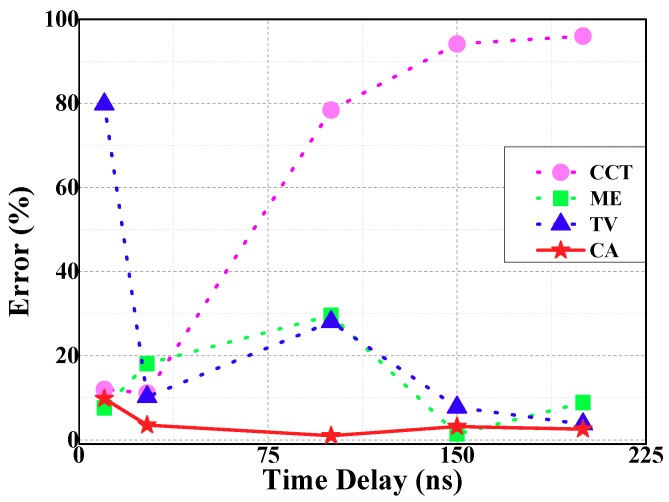
Extraction algorithm of TDoA analysis and comparison.

For this individual case of GIS, 55 ns was set as the threshold. If the TDoA of the ME is 55 ns and below, the TDoA acquired by ME and CCT will be processed on average and output the value. Otherwise, this value is the average TDoA of ME and TV. The error of CA and three typical algorithms is shown in Figure 12.

The coincidence degree between CA and reference TDoA curve is the highest. By calculating, the average error of CA is 4.0% and the variance is 0.00092. The accuracy of TDoA extraction can be improved using CA.

## 6. Conclusions

Based on the real insulation fault of GIS, the definition of the first wave had been clarified. The TDoA between OCU 7 and OCU 11 UHF signals extracted by the oscilloscope is used as an example. Furthermore, through a series of analyses, the following conclusions are obtained:

(1) CCT is suitable for the application of short TDoA; ME has low average error and wide application range, but the TDoA is usually less than the reference TDoA; TV is greatly influenced by the selection of the threshold value. It is different from the CCT that TDoA extracted by the TV is generally greater than the reference TDoA.

(2) CA is based on the minimum energy method. For the GIS equipment involved in this paper, 55 ns was taken as the threshold after many tests and the value might be variable as to other GIS equipment. If the TDoA of the ME is less than 55 ns, the TDoA is average of ME and CCT. Otherwise, the TDoA is the average of ME and TV. The average error of CA is 4.0% and the variance is 0.00092. The extraction accuracy of TDoA is improved significantly.

## Figures and Tables

**Table 1:** Time Differences of Arrival (TDoA) extracted by typical methods.

OCU Number		Cross-Correlation Function (CCF)	Minimal Energy (ME)	Threshold Value (TV)
		TDoA (ns)	TDoA (ns)	TDoA (ns)
a	b	8.80	9.24	17.98
7	8	24.00	31.89	29.75
7	11	21.58	70.39	128.00
8	22	8.80	147.92	161.60
8	18	8.00	182.30	207.5

**Table 2:** Extraction algorithm of time difference analysis and comparison.

Reference TDoA (ns)	Cross-Correlation Function (CCF)			Minimal Energy (ME)			Threshold Value (TV)		
	TDoA (ns)	Error (%)	Variance	TDoA (ns)	Error (%)	Variance	TDoA (ns)	Error (%)	Variance
10	8.80	12.00	0.15	9.24	7.60	0.01	17.98	79.80	0.08
27	24.00	11.11		31.89	18.11		29.75	10.19	
100	21.58	78.42		70.39	29.61		128.03	28.03	
150	8.80	94.13		147.92	1.39		161.60	7.73	
200	8.00	96.00		182.30	8.85		207.5	3.75	
